# Role of sodium/glucose cotransporter inhibition on a rat model of angiotensin II–dependent kidney damage

**DOI:** 10.1186/s12882-019-1490-z

**Published:** 2019-08-02

**Authors:** Humberto Reyes-Pardo, Rocío Bautista, Hilda Vargas-Robles, Amelia Rios, Daniel Sánchez, Bruno Escalante

**Affiliations:** 1 Unidad Monterrey, Centro de Investigación y de Estudios Avanzados del Instituto Politécnico Nacional, Vía del Conocimiento 201, PIIT, N.L, 66600 Apodaca, Nuevo León Mexico; 2 Department of Nephrology, Instituto Nacional de Cardiología “Ignacio Chávez”, México City, Mexico; 3 Department of Molecular Biomedicine, Centro de Investigación y de Estudios Avanzados del Instituto Politécnico Nacional, México City, Mexico; 4 Basic Science Department, Monterrey University, Morones Prieto 4500, 66238 San Pedro Garza Garcia Nuevo León, N.L. Mexico

**Keywords:** SGLT2, Angiotensin II, ROS, Kidney damage

## Abstract

**Background:**

Renal proximal tubular sodium and glucose reabsorption are regulated by the sodium-glucose cotransporter (SGLT2). Changes in this transporter can play a role in hyperglycaemia and reactive oxygen species (ROS) production. We demonstrated increased glucose absorption in proximal tubule membrane vesicles and increased expression of SGLT2 in hypertensive rats. Here we investigated Angiotensin II (Ang II) -dependent SGLT2 expression induction and the role of SGLT2 induction in the development of Ang II-dependent kidney damage. The aim of this study was to determine whether SGLT2 induction by Ang II is associated with Ang II-dependent kidney damage. We propose the following objectives a) to demonstrate that Ang II induces SGLT2 expression and b) to demonstrate that prevention of SGLT2 expression and activity prevent Ang II-induced kidney damage.

**Methods:**

We used chronic Ang II infusion as a model of kidney damage in male Wistar rats and evaluated systolic blood pressure by telemetric methods. SGLT2 mRNA and protein expression were evaluated by PCR and immunoblotting. SGLT2 activity was evaluated in brush border membrane vesicles by measuring glucose uptake. ROS production was measured by confocal microscopy. The glomerular filtration rate (GFR) was evaluated by the inulin excretion method, and urinary protein excretion was evaluated by the Bradford method. Biological parameter evaluations were performed, after two weeks of infusion of Ang II. We compared the effects of Angiotensin II (AT1) receptor blockade by Losartan and SGLT2 inhibition by Empagliflozin both as monotherapy treatments and in combination on the development of kidney damage.

**Results:**

Chronic Ang II infusion led to a blood pressure elevation and increased SGLT2 mRNA expression and activity as well as kidney damage, as reflected by increased ROS production, decreased GFR and increased urinary protein excretion. AT1 receptor blockade prevented all these changes. By contrast, SGLT2 inhibition did not affect blood pressure and had a small effect on kidney damage. However, the combination of both drugs resulted in the potentiation of the effects observed by AT1 receptor blockade alone.

**Conclusions:**

We suggest that Ang II-dependent increased SGLT2 induction is one mechanism by which Ang II induces kidney damage.

**Electronic supplementary material:**

The online version of this article (10.1186/s12882-019-1490-z) contains supplementary material, which is available to authorized users.

## Background

Hypertension is a high-risk factor for cardiovascular and renal diseases. Studies in normotensive or hypertensive patients have shown a correlation between sodium intake and blood pressure (BP) [1]. Renal proximal tubular sodium reabsorption is regulated by sodium transporters, including sodium glucose cotransporters (SGLTs), sodium amino acid transporter, sodium hydrogen exchanger, sodium phosphate cotransporter, and sodium potassium ATPase. Recently, it has been reported that changes in these transporters play a role in the increase of BP in essential/polygenic hypertension [2]. The SGLTs encompass a family of membrane proteins responsible for the transport of glucose and sodium across the brush border membrane of renal proximal tubules. SGLT2, exclusively expressed in the S1 and S2 segments of the renal proximal tubule [3], mediates sodium and glucose cotransport in a 1:1 stoichiometry and has been proposed to be responsible for 60–90% of renal sodium/glucose uptake [4]. Concurrent with the characterization of SGLTs in the 80s and 90s, there has been increased interest in developing SGLT2 inhibitors as novel mechanisms for reducing hyperglycaemia [5]. In addition to the hypoglycemic effect of SGLT2 inhibition, several studies have related SGLT2 inhibitors to the reduction of hypertension. Thus, the use of these new SGLT2 inhibitors has been shown, as a monotherapy or an add-on to another hypoglycemic drug, to reduce systolic and diastolic BP without changing in heart rate or increasing episodes of fainting [6, 7]. In a recent study using dapagliflozin, patients with increased BP receiving their usual standard antihypertensive treatment, showed a decrease in mean seated systolic and diastolic BP. A post-hoc analysis of patients with a history of hypertension and who did not meet the BP goal of less than 130/80 mmHg at baseline showed that 29.5–37.5% of such patients assigned to dapagliflozin achieved the goal at week 24 compared with 8.8% of patients assigned to placebo [8]. These results suggest that SGLT2 may be associated with the mechanisms of hypertension development. We reported increased glucose absorption in proximal tubule membrane vesicles and increased expression of SGLT2 in response to renovascular hypertension in rats. We postulated that Angiotensin II (Ang II)-dependent induction of SGLT2 may be associated with increased Na^+^ reabsorption and this regulation may have important physiological and/or physiopathological consequences [9]. However, in addition to the vasoconstrictive, prohypertensive and sodium reabsorption effects associated with Ang II, proinflammatory mechanisms have been described [10]. Indeed, Ang II induced hypertension and increased chemokines and reactive oxygen species (ROS), which may be major contributors to the development of kidney failure [11].

We have previously shown the relationship between SGLT2 and oxidative stress in renal tissue [12]. Thus, the possibility arises that Ang II kidney damage caused by reactive oxygen species may be related to SGLT2 expression. Recent studies have suggested that the beneficial effects of SGLT2 inhibition on kidney protection are probably related to the reduction of renal inflammation and ROS production [13] or through decrease of glomerular hyperfiltration, by reducing tubuloglomerular feedback [14].

In the present study, we hypothesized that Ang II increases the inflammatory process through regulation of proinflammatory messengers and by increasing hyperglycaemic proinflammatory mechanisms through increased SGLT2 expression. This hypothesis is supported by the suggestion that Ang II system inhibition in combination with SGLT2 inhibitors may be a good therapeutic strategy to prevent kidney damage associated with Ang II [15]. Thus, in the present study, the principal objective is to demonstrate that SGLT2 induction by Ang II is associated with Ang II-induced kidney damage. We propose as secondary objectives a) to demonstrate that increased kidney SGLT2 expression is Ang II-dependent and b) to demonstrate that increased SGLT2 activity is associated with the development of kidney damage. The use of the rat model for Ang II-induced kidney damage allows the exploration of the cellular mechanisms involved in the therapeutic effects of SGLT inhibition and AT1 receptor blockade as mono- or combination therapy, supporting a clinical use of either therapy. Therefore, in the present study, we compared the use of an Ang II Type 1 receptor blocker (Losartan), SGLT2 inhibition (Empagliflozin) or the combination of both treatments on the Ang II-dependent effects on kidney damage in rats.

## Methods

**Fig. 1:**
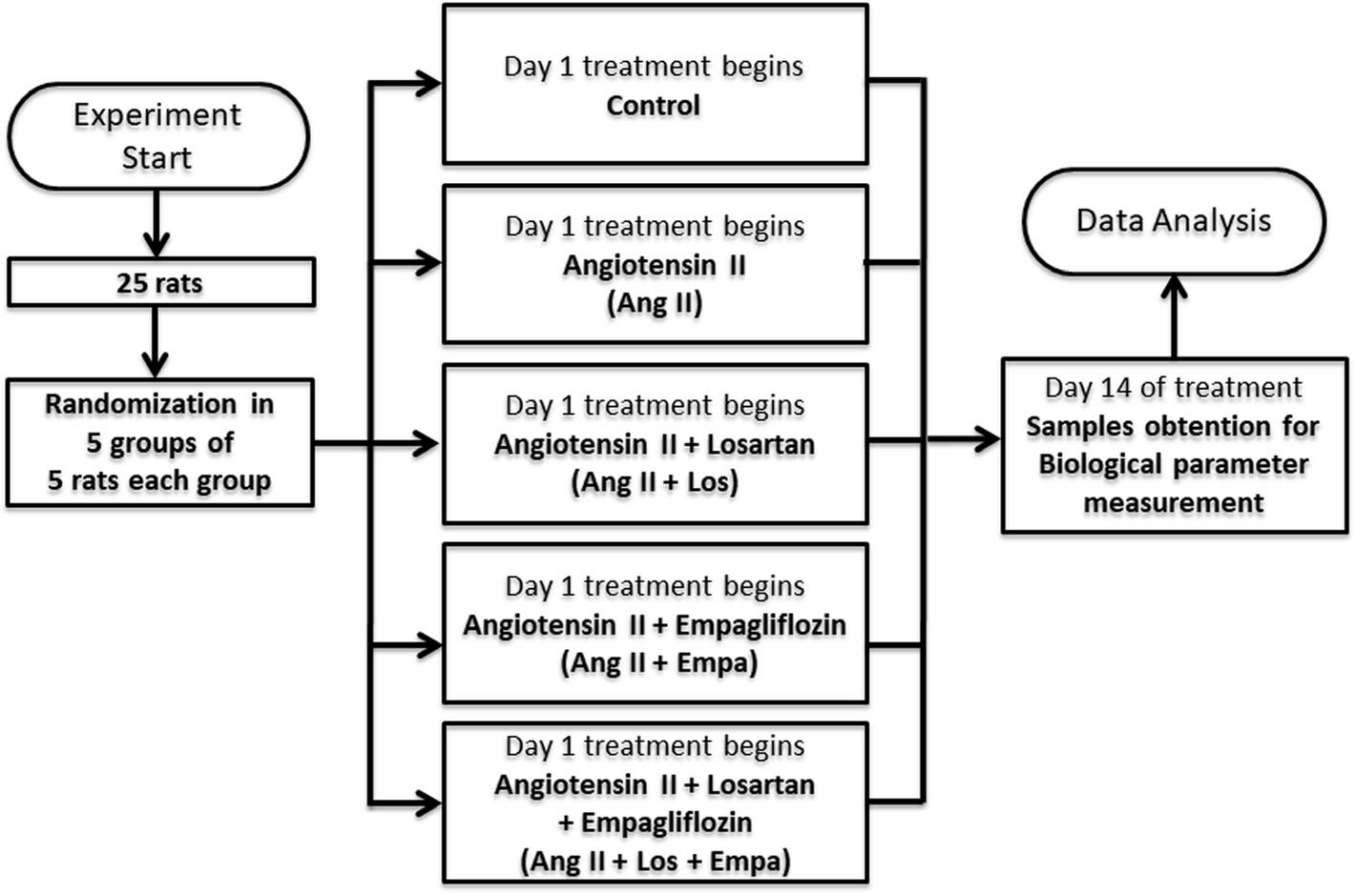
The flowchart of animal experiments. Number of animals used in each experiment and treatment groups. On day 1 treatment was started in each group and on day 14 the samples were obtained for the measurement of a specific biological parameter. This flowchart of the process was repeated for the measurement of each specific biological parameter

We used a total of 125 male Wistar rats 140 ± 3 days old with a weight of 300–375 g. Rats obtained from the CINVESTAV animal care facility were bred and housed on pathogen filtered cages, 12 h light/dark cycles, food (PicoLab Rodent Diet 20, LabDiet, PMI Nutrition International LLC. Brentwood, MO) and water ad libitum, room was maintained under weather (25 °C) conditions as well as pathogen control. Experiments were approved by the professors of the animal care committee of CINVESTAV. Rats were randomly divided into 5 different experimental groups (2 rats per cage): a) rats without treatment (Control), B) rats receiving infusion of 400 ng/kg/min Ang II (Ang), C) rats receiving Ang II infusion plus 1 mg/kg/day Ang II Type1 receptor blocker Losartan (Los), D) rats receiving Ang II plus 10 mg/kg of SGLT2 inhibitor Empagliflozin (Emp) and E) rats receiving Ang II plus the combination of Losartan and Empagliflozin (Los + Emp), each experimental group consisted of 25 rats and was obtained from different littermates, Losartan and Empagliflozin treatment were administered by gavage twice a day. Because several of the measurements performed required rat sacrifice or the use of both kidneys, each of the above-mentioned experimental groups was subdivided to evaluate 1) blood pressure, 2) ROS production, 3) glomerular filtration rate (GFR), 4) SGLT2 mRNA and protein levels, and 5) glucose uptake. Each of this subgroup consisted of 5 rats. Treatment, experimental procedure and data analysis were performed independently for each group and then the next group experiments were started. According to the survival rate for the experimental manipulations, a maximum of 7 rats was used to have a final n of 5 rats for each group (Fig. 1). We have previously shown clear differences between the control and treated groups using *n* = 5 [9, 12, 16, 17], therefore, to use the minimum number of animals, we decided to use groups of 5 rats. All groups and subgroups were randomly divided, and the technician in charge of the biological measurements was not aware of the treatments. Ang II infusion as well as Losartan, Empagliflozin or combination treatment were simultaneously initiated immediately after Ang II infusion was started and lasted 14 days. Either Angiotensin II pump implantation or oral treatment were performed in treatment room with similar characteristics to the breeding area. For chronic Ang II infusion, Alzet mini-osmotic pumps (model 2001, Alzet, Palo Alto CA) with a capacity of 200 ± 10 μl and an infusion rate of 1.0 ± 0.15 μl/h were filled with Ang II (Sigma Aldrich, St Louis, MO) to obtain an infusion rate of 400 ng/kg/min [16]. Blood pressure was monitored during the 14 days of treatment. At the end of the treatment period, urinary protein excretion, GFR, SGLT2 mRNA and protein levels, glucose uptake and renal in situ ROS production were evaluated. At the end of the 14 days of treatment, rats were anesthetized with pentobarbital sodium (50 mg/kg, i.p.), and the kidneys were quickly perfused and removed to isolate brush-border membrane vesicles (BBMV) and perform immunohistochemical studies and RT-PCR analysis. Following the extraction of kidneys, the rats were sacrificed using an overdose of pentobarbital sodium (150 mg/kg, i.p.).

### Blood pressure measurements

One day before Ang II or other treatments started, 5 Wistar rats 142 ± 3 days old, with a weight of 375 ± 30 g, were anesthetized and subjected to implantation of an intraperitoneal device (TA11PA-C40) for telemetric measurements of blood pressure according to the manufacturer’s instructions (Data Sciences International). Blood pressure was measured daily for a period of 10-min for 14 days. However, only the results for days 0, 1, 3, 7 and 14 are presented. The recorded blood pressure measurements were saved for further analysis [17].

### Urinary protein excretion

At the end of the 14 days, 5 Wistar rats 143 ± 3 days old, with a weight of 275 ± 30 g, were placed in metabolic cages for 24 h to collect urine samples, and urinary protein was measured by the Bradford method [18], Sample replicates were diluted 1:100 and read at 595 nm in a Beckman DU-650 spectrophotometer. Final values represent the means of 5 different rats. A standard curve was prepared with bovine serum albumin.

### Glomerular filtration rate

At day 14, 5 Wistar rats 140 ± 3 days old, with a weight of 340 ± 20 g, were anesthetized and GFR was measured by a bolus injection of 3 μl/g of body weight of fluorescein isothiocyanate–inulin (inulin-FITC) (Sigma-Aldrich, St. Louis, MO, USA), followed by 30 min constant infusion of 0.15 μl/min/g of body weight of inulin-FITC. At the end of stabilization urine was collected for 30 min. Blood samples (100 μl) were taken before and after urine collection. Fluorescence of 3 replicates each of urine and blood samples was measured with a fluorescent microplate reader (Labsystems Fluoroskan, Labsystems Diagnostics OY, Helsinki, Finland), excited at 485 nm and the emission read at 538 nm. The ratio of urine fluorescence X urine volume/blood fluorescence X collection time was calculated and corrected by kidney weight expressed as μl/g kidney weight. Each value reported represents the means of 5 different rats.

### SGLT2 mRNA expression

At day 14, 5 Wistar rats 144 ± 4 days old, with a weight of 355 ± 20 g, were anesthetized and both kidneys were extracted. The renal cortex was obtained from all experimental groups, and total RNA was isolated using TRIzol reagent (Gibco BRL, Life Technologies, Waltham, MA, USA). Samples of 2 μg of total RNA were reverse transcribed to cDNA using the Superscript II RNAse H-Reverse Transcriptase Kit (Gibco BRL, Life Technologies, Waltham, MA, USA). PCR was carried out using a Perkin Elmer Gene Amp 2400 PCR system performing a 35 cycles protocol at 94 °C for 1 min, 64 °C for 1 min and 72 °C for 1 min, followed by a 10 min extension period at 72 °C. The primers used for SGLT2 amplification were: sense, 5′-ccaatagaggcacagttggtgg-3′ and antisense, 5′-cgtaaatgttccaacgg-3′ and for GAPDH amplification: sense, 5′-ggatttggccgtattggcc-3′ and antisense, 5′-catgtccagatcacaacgg-3′ [9].

### SGLT2 protein expression

Immunoblotting analysis was used for the SGLT2 cotransporter, samples from BBMV for the experimental groups or LLC-PK_1_ cultured cells incubated during 15 min with increasing Angiotensin II (10^-11^ to 10^-7^ M) concentrations, were washed, and cell suspension homogenized in ice cold proteinase-inhibitor containing NP40-supplemented lysis buffer with the following composition: 20 mM Tris-HCl, pH 7.5, 100 mM NaCl, 10 mM MgCl_2_, 1 mM EDTA, 1 mM EGTA, 0.5 mM NP40, 2.5 mM Na_4_P_2_O_7_, 1 mM b-glycerophosphate, 1 mM Na3VO4, 25 mM NaF, 1 mg/ml leupeptin, 1 mg/ml aprotinin,1 mM phenylmethylsulphonyl fluoride and 1 mM dithiothreitol. The detergent-containing extract was then cleared by centrifugation (15,000 g for 10 min). Total protein was determined by Biorad protein assay (Bio-rad, Mississauga ON, Canada). 10 μg of protein for each sample are denatured at 92 °C for 6 min in Laemmli buffer and resolved on 4-20% Tris-Glycine gels, run at 120 V for 2 h. Proteins were transfer to PVDF membrane, blocked for 1 h at room temperature in PBS with 0.1% Tween-20 containing 1% ECL blocking agent (GE Healthcare, Waukesha WI, USA), and incubated overnight at 4 °C with SGLT2 antibody (11000) (Alpha Diagnostic International, San Antonio TX, USA) diluted in blocking solution. Blots were stained for horse radish peroxidase activity using the enhanced chemiluminescence detection system (ECL Kit, Amersham Pharmacy Biotech, Piscataway NJ, USA). After detection, samples were measured by densitometry with a Kodak electrophoresis documentation and analysis system (EDAS 290. Kodak, Rochester NY, USA) (9).

### Glucose uptake

At day 14, 5 Wistar rats 145 ± 5 days old, with a weight of 345 ± 30 g, were anesthetized and both kidneys were extracted. Na^+^-Glucose cotransporter activity was evaluated by measuring glucose uptake in brush border membrane vesicles (BBMV) as previously reported [9], BBMV were prepared following the two-step precipitation method with MgCl_2_. Briefly the renal cortices from 2 rats from each experimental group were macerated and suspended in mannitol hypotonic buffer (10 mM mannitol, 2 mm Tris-H_2_SO_4_, pH 7.4) and subsequently homogenized at 20500 rpm for 2 min with a polytron (Ultra-Turrax T-25, IKA Labortechnik, Staufen, Germany). The homogenates were subjected to 2 cycles of precipitation with 10 mM MgCl_2_ and centrifugation (Sorvall RC-5B. DuPont Co. Wilmington, DE, USA). Between the precipitation steps, the pellet was resuspended and homogenized with 10 strokes of a Dounce glass pestle homogenizer. Finally, the vesicles formed were resuspended in the intravesicular solution containing 100 mM mannitol, 100 mM KCl and 20 mM HEPES/Tris. The BBMV were kept frozen using liquid nitrogen until they were used. Glucose transport was measured by a rapid filtration technique. Glucose uptake was started by combining 10 μl (3 mg/ml) of the BBMV with 50 μl of Glucose uptake medium containing 10 μCi/ml ^3^H-labeled glucose (Amersham Life Science), 100 mM mannitol, 100 mM KCl and 10 mM HEPES/Tris. The reaction was stopped at 5 min by diluting the mixture with 1 ml of ice-cold stop solution containing 300 mM mannitol, 80 mM Na_2_SO_4_, 10 mM HEPES/Tris-H_2_SO_4_, 0.3 mM Phlorizin pH 7.4. This solution was filtered immediately through 0.65 μm pore size wet Millipore filters and kept under negative pressure. The filters were washed twice with 1 ml stop solution and dissolved in 5 ml scintillation fluid (Aquasol-2, PerkinElmer Inc., Wellesley, MA, USA). Experiments were performed at room temperature using 3 replicates for each measurement. Radioactivity was measured with a scintillation counter (Beckman LS6500) and the non-specific binding to the filters was corrected with samples in the absence of BBMV. The data obtained are expressed in picomoles per milligram per 15 s.

### Renal in situ ROS production

At day 14, 5 Wistar rats 140 ± 4 days old, with a weight of 387 ± 25 g, were anesthetized and both kidneys were extracted. Renal in situ production of ROS was observed by oxidative fluorescence microphotography. Dihydroethidium (DHE), an oxidative fluorescence dye, was used to assess the production of ROS in situ, as previously described [19]. Briefly, unfixed tissue samples were frozen and cut into 10 μm thick slices and laid on microscope glass slides. Tissue slices were incubated with or without superoxide dismutase-polyethylene glycol (PEG-SOD, 120 U/ml., Sigma Aldrich, St. Louis, MO, USA) for 60 min. 10 μM DHE (Molecular Probes, Thermo Fisher Scientific, Waltham, MA, USA) was added to each tissue section before placing the coverslips. The samples were incubated for 30 min in the dark at 37 °C in a humidified chamber. DHE fluorescence images were acquired with a confocal microscope system (FV-300, Olympus America, Melville, NY, USA), exciting the sample at a wavelength of 488 nm and capturing the fluorescence emission at 610 nm. Generation of superoxide was identified by SOD inhibition in the red fluorescence labelling of tissue samples.

### Statistics

All results are expressed as the means ± SEM. Significance was determined by a two-factor repeated measures ANOVA. A significant F-test from the ANOVA at *P* < 0.05 was followed by a Post Hoc comparison using Newman-Keuls multiple range test with a significance of *P* < 0.05.

## Results

The health status of the used rats, prior to the experimental treatment, was good. Basic biological parameters monitored before Ang II or pharmacological treatment was not different between the experimental groups as shown in Table 1.

**Table 1:** Baseline biological parameters previous Ang II or pharmacological treatment

Group	Sex	Age (weeks)	Weight (g)	Blood pressure (mmHg)	Urinary volume (ml/Day)	Urinary protein (mg/Day)
Control	Male	20	360 ± 7	112 ± 0.7	16 ± 1.0	7.30 ± 0.4
Ang II	Male	20	355 ± 3	109 ± 1.5	15 ± 1.2	7.25 ± 0.2
Ang II + Los	Male	20	358 ± 5	109 ± 1.1	17 ± 1.5	7.60 ± 0.4
Ang II + Empa	Male	20	363 ± 5	110 ± 1.2	14 ± 1.0	7.14 ± 0.3
Ang II + Los + Empa	Male	20	359 ± 4	111 ± 1.5	18 ± 1.9	7.21 ± 0.2

### Blood pressure

**Fig. 2:**
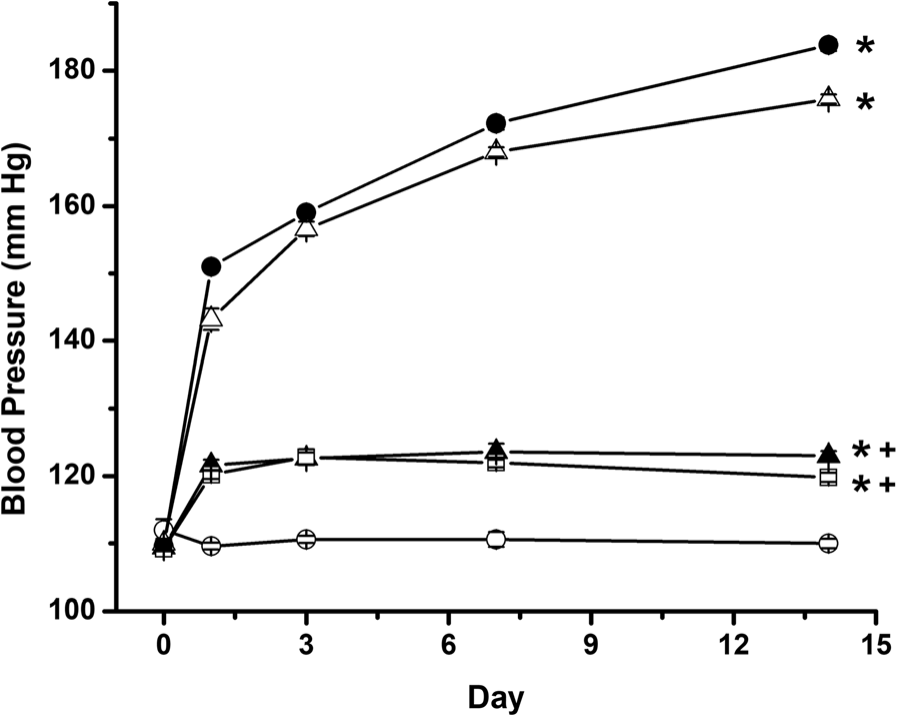
Blood Pressure time course in rats infused for 2 weeks with vehicle (○), Ang II (●), Ang II plus Losartan treatment (▲), Ang II plus Empagliflozin treatment (△) and Ang II plus the combination of Losartan and Empagliflozin treatments (□). Each bar represents the mean ± SE of 5 different rats for each experimental group, *p < 0.05 compared vs vehicle, **+** *p* < 0.05 compared vs Ang II

Chronic Ang II infusion induced a time-dependent increase in blood pressure (Fig. 2). After 14 days of Ang II infusion, blood pressure was significantly higher (183.8 ± 0.8 mmHg) (*n* = 5) compared to control (110 ± 0.7 mmHg) (*n* = 5) rats. Ang II Type 1 receptor blockade by Losartan treatment prevented blood pressure increase; thus, 14 days after Losartan treatment, the blood pressure value was 123 ± 0.7 mmHg (n = 5). SGLT2 inhibition by Empagliflozin did not affect Ang II induced-hypertension, and the blood pressure value was 175.8 ± 0.7 mmHg (*n* = 5). Combination of Losartan and Empagliflozin prevented Ang II-induced hypertension. However, blood pressure value of 119.8 ± 0.6 mmHg (*n* = 5) were not different compared with those observed with Losartan treatment alone (Fig. 2).

### SGLT2 mRNA and protein expression

**Fig. 3:**
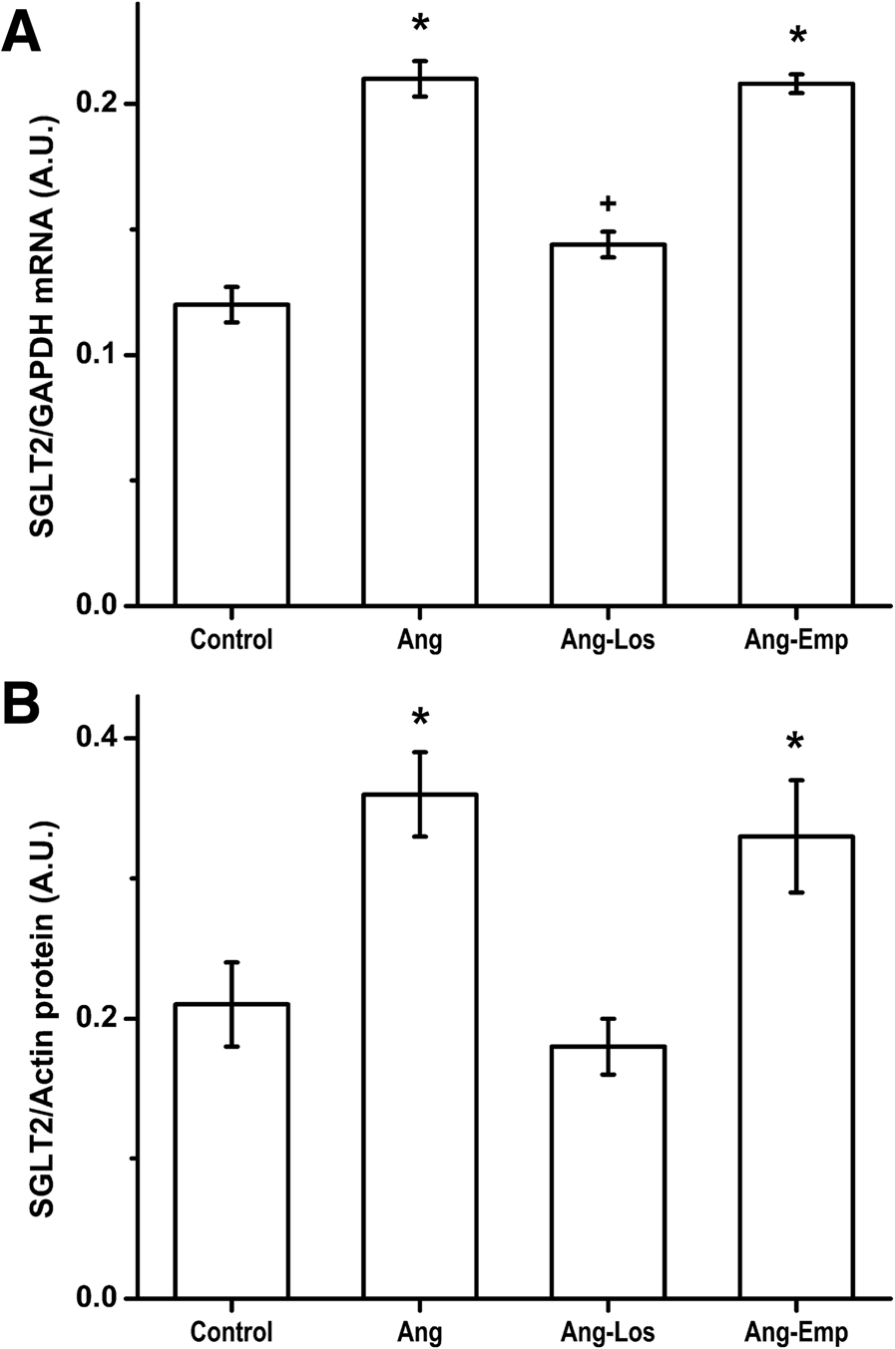
Sodium-glucose cotransporter mRNA expression (**a**) and Immunoblotting (**b**) in rats infused for 2 weeks with vehicle (Control), Ang II (Ang), Angiotensin II plus Losartan treatment (Los) and Ang II plus Empagliflozin treatment (Emp). Each bar represents the mean ± SE of 5 different rats for each experimental group, * *p* < 0.05 compared vs vehicle, **+** *p* < 0.05 compared vs Ang II

Ang II infusion for 14 days resulted in a significant increase in SGLT2 mRNA and protein expression in the renal tissue compared to control rats. Losartan alone or in combination with Empagliflozin partially prevented Ang II-induced SGLT2 mRNA and protein expression increase. By contrast Empagliflozin treatment did not affect the increase in SGLT2 mRNA and protein expression induced by Ang II infusion (Fig. 3) (*n* = 5 in each treatment group). We also observed that cultured epithelial cells incubated with increasing Ang II concentrations (0.01, 0.1, 1, 10 and 100 nM), exhibited increased SGLT expression, at 0.15 ± 0.01 on control to 0.16 ± 0.007, 0.25 ± 0.01, 0.23 ± 0.01, 0.23 ± 0.01 and 0.15 ± 0.009 (SGLT2/GAPDH in arbitrary units), respectively (Additional file 1: Figure S1).

### Sodium glucose cotransporter activity

**Fig. 4:**
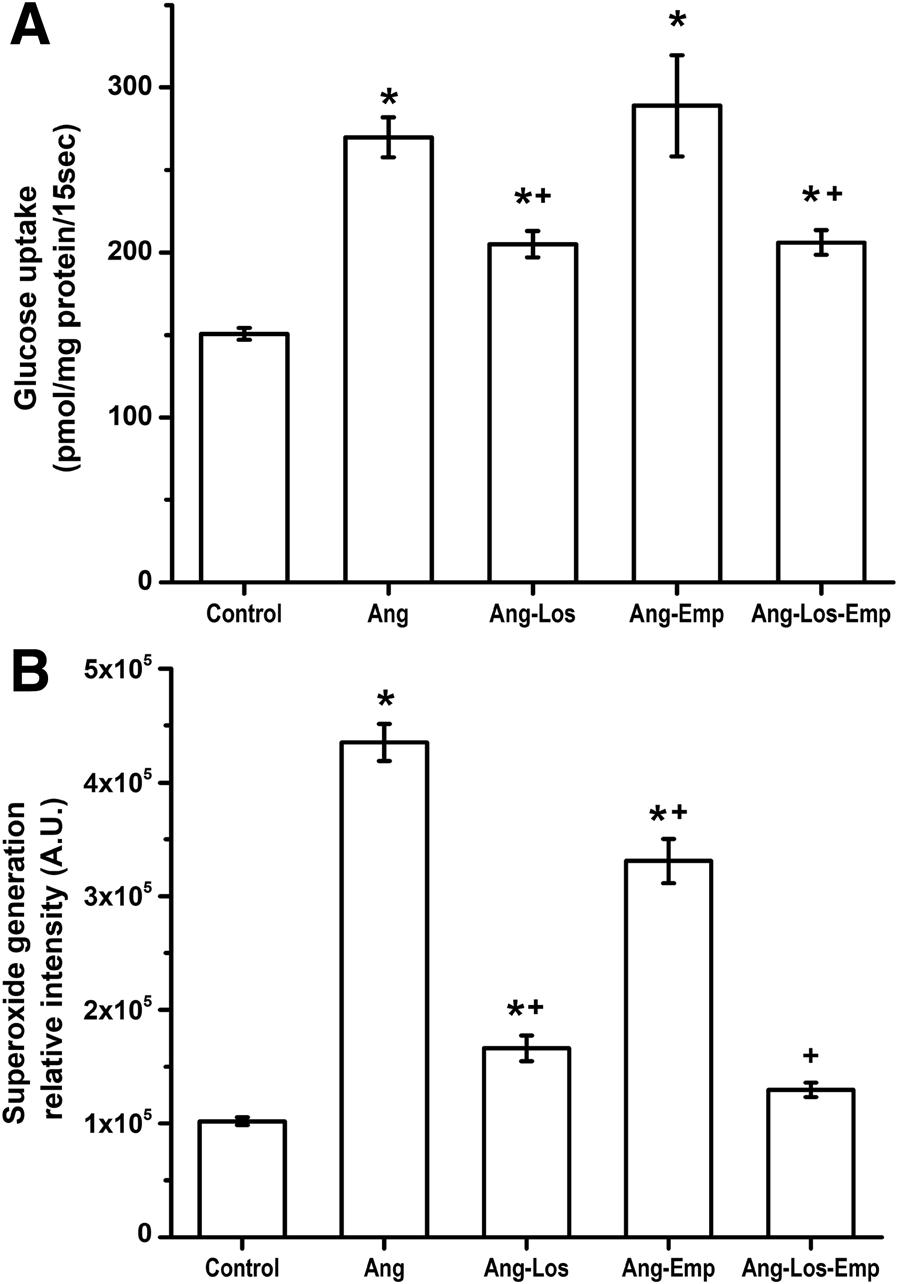
Glucose Uptake (**a**) and representative immunohistochemical staining for Reactive Oxygen Species (**b**) in rats infused for 2 weeks with vehicle (Control), Angiotensin II (Ang), Ang II plus Losartan treatment (Ang-Los), Ang II plus Empagliflozin treatment (Ang-Emp) and Ang II plus the combination of Losartan and Empagliflozin treatment (Ang-Los-Emp). In B, the graph represents semiquantitative analysis of fluorescence intensity of the immunohistochemical staining. Each bar represents the mean ± SE of 5 different rats for each experimental group, *p < 0.05 compared vs vehicle, **+** *p* < 0.05 compared vs Ang II

SGLT2 activity in proximal tubule brush borders from Ang II-infused rats was increased, as demonstrated by increased glucose uptake (Fig. 4a) (*n* = 5 in each treatment group). Thus, glucose uptake increased in comparison to control rats. This increase induced by Ang II was prevented by Losartan and by the combination of Losartan plus Empagliflozin treatment. Interestingly, brush border membranes from Empagliflozin-treated rats had a glucose uptake similar to the values observed in the Ang II rats (Fig. 4a) (*n* = 5 in each treatment group).

### Reactive oxygen species generation

ROS in the renal tissue from rats that received Ang II infusion was significantly increased by 4.4–fold compared with control rats (Fig. 4b) (n = 5 in each treatment group). This increase in ROS was prevented by Losartan, Empagliflozin and Losartan plus Empagliflozin combination treatment. However, prevention by the combination of both treatments was higher than the effect produced by either treatment alone. Prevention of the Ang II-induced ROS increase with these three treatments was 59 ± 1.7%, 24 ± 6.4% and 72 ± 0.8% respectively (*n* = 5 for each treatment group).

### Glomerular filtration rate

**Fig. 5:**
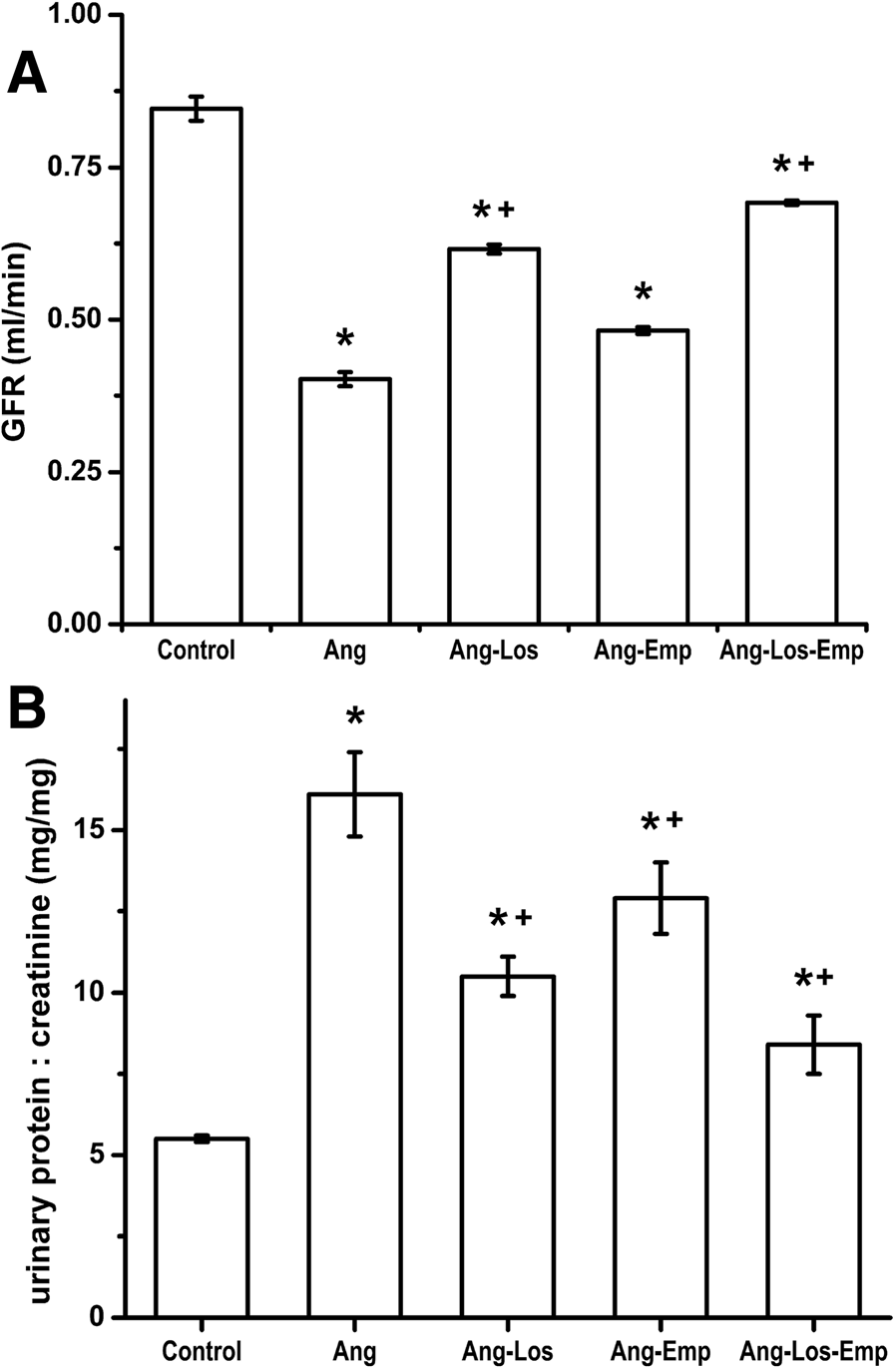
Glomerular Filtration Rate (**a**) and Urinary Protein Excretion (**b**) in rats infused for 2 weeks with vehicle (Control), Ang II (Ang), Ang II plus Losartan treatment (Ang-Los), Ang II plus Empagliflozin treatment (Ang-Emp) and Ang II plus the combination of Losartan and Empagliflozin treatment (Ang-Los-Emp). Each bar represents the mean ± SE of 5 different rats for each experimental group, *p < 0.05 compared vs vehicle, **+** *p* < 0.05 compared vs Ang II

Fourteen days of Ang II infusion significantly reduced GFR compared with control rats (Fig. 5a) (n = 5 in each treatment group). Both Losartan and Empagliflozin monotreatment prevented the effect of Ang II on the GFR. Moreover, the combination of Losartan and Empagliflozin resulted in a better prevention on the Ang II effect on the glomerular filtration rate. Prevention of the Ang II-induced GFR reduction was 47 ± 3.3%, 19 ± 2.4% and 66 ± 2.8% respectively, in these groups (*n* = 5 for each treatment group).

### Urinary protein excretion

As illustrated in Fig. 5b, Ang II infusion significantly increased urinary protein excretion. This value was higher compared with that of the control rats (43 ± 2.1 vs 7.4 ± 0.2 mg/day n = 5 in each group). Losartan and Empagliflozin monotreatment prevented increased urinary protein excretion induced by Ang II. Furthermore, combination of Losartan and Empagliflozin treatment resulted in a better prevention of the Ang II effect on urinary protein excretion. Prevention of the Ang II-induced urinary protein excretion increase in these three treatment groups was 59 ± 4.7%, 33 ± 6.6% and 71 ± 2.7% respectively (*n* = 5 in each treatment group).

## Discussion

Elevated intrarenal activity of the renin-angiotensin system may be an important determinant of sodium retention and BP as well as kidney damage, and this effect may be associated with increased tubular SGLT2 expression. In this study we evaluated the effect of elevated Ang II concentration on SGLT2 expression and the effect of this increased SGLT2 expression on BP and kidney damage by comparing prevention of the Ang II-induced kidney damage by either AT1 receptor blockade (Losartan), SGLT2 inhibition (Empagliflozin) monotherapy or by the combination of both treatments. Previously we have shown increased SGLT2 expression in renal-dependent hypertensive or diabetic animals. This increased expression was blocked by treatment with an AT1 receptor antagonist, suggesting that the renin-angiotensin system may act locally in the tubular cells and modulate glucose transporter gene expression [9, 20]. In the present study we confirmed our previous results by showing that a direct increase in systemic Ang II was associated with increased SGLT2 mRNA expression or activity. Evidence of this conclusion is supported by the observation that both effects were clearly blocked in the presence of the AT1 receptor blocker Losartan. This further supports our hypothesis that Ang II is responsible for SGLT2 gene regulation, as suggested by the initial indirect evidences previously reported by our group [21]. This idea is further supported by our data in cultured cells showing Ang II-dependent SGLT2 expression. Up-regulation of Ang II and sodium glucose cotransporter at an early age during nephrogenesis also suggests a relationship between Ang II and SGLT2 expression [22]. Furthermore, in vivo Ang II administration has been associated with an effect on proximal cell SGLT2 expression [23]. These data support previous results demonstrating that Ang II or Ang II antagonists participate in transport protein regulation [24, 25].

Induction of SGLT2 gene expression observed in the renal cortex from Ang II treated rats in this study may reveal an important physiological or pathophysiological mechanism associated with Ang II-dependent development of hypertension and/or kidney damage. The consequences of this induction not only represent responses designed to adjust the trans-tubular glucose in different filtration rates but also play an active role in renal injuries. A physiological defect in sodium excretion has been suggested as the mechanism involved in the kidney to cause hypertension [26]. Several forms of genetic hypertension associated with enhanced sodium reabsorption support the idea that hypertension results from alterations in the regulation or expression of tubular transport systems involved in sodium transport [27]. However, our data suggest that increased SGLT2 induction by Ang II plays no role in hypertension development, as demonstrated by the observation that SGLT2 inhibition by Empagliflozin did not affect hypertension development during Ang II infusion. This conclusion contradicts several clinical studies that have related SGLT2 to hypertension. Thus, SGLT2 inhibition decreased systolic and diastolic BP [6, 7]. Moreover, combination of SGLT2 inhibition with anti-hypertensive drugs increased the number of patients meeting blood pressure goals of less than 130/80 mmHg [8]. Weight loss and a slight diuretic effect have been described with the use of SGLT2 inhibitors and this can be related to the anti-hypertensive effect of SGLT2. In the present study, the lack of anti-hypertensive effects of SGLT2 inhibition may be explained by the observation that neither weight lost nor glucosuria were observed in the presence of SGLT2 inhibition.

However, despite the lack of a hypertensive effect of SGLT2 inhibition during Ang II infusion, we observed that Empagliflozin prevented ROS generation, proteinuria and GFR reduction associated with Ang II infusion, reducing kidney damage development. Several mechanisms for Ang II renal damage have been described. First, it is known that Ang II-dependent kidney damage can be independent of blood pressure elevation [28]. Thus, our data may suggest that prevention of kidney damage by SGLT2 inhibition may be associated with additional protective mechanisms of action. Ang II-dependent kidney damage can be related to ROS production [29]. In the present study we have demonstrated that Ang II induced ROS production was prevented by SGLT2 inhibition, suggesting the possibility that SGLT2 induction and ROS by Ang II are part of the same mechanism associated with the development of stress renal tissue damage by Ang II. Second, increased GFR represents a mechanism that has been proposed to be part of the kidney damage development by Ang II. [30]. Our data show that SGLT2 inhibited prevention of Ang II-dependent decrease in GFR. This observation further supports our hypothesis that, induction of SGLT2 expression by Ang II may represent one of the pathophysiological mechanisms elicited by Ang II to induce kidney damage. Furthermore, the question that arises is how relevant is this SGLT2-dependent mechanism to the Ang II-induced kidney damage. Our results suggest that SGLT2 represents a complimentary or secondary mechanism in the Ang II-induced kidney damage. We support our hypothesis by the following observation: increased blood pressure due to Ang II was not affected by SGLT2 inhibition; however, Ang II induced kidney damage is partially prevented by SGLT2 inhibition, suggesting two different sites of action for the SGLT2 inhibition. Prevention of kidney damage was different, and the AT1 receptor blockade effect was 55% compared to that produced by SGLT2 inhibition of 25%. Moreover, the combination of both treatments increased kidney damage prevention. Therefore, the additive effect observed by the addition of Empagliflozin to Losartan treatment supports the idea that both systems are independent and affecting different mechanisms. Indeed, it has been suggested that SGLT2 inhibition by increasing Na^+^ delivery to the macula densa is increasing GFR, thereby protecting kidney function [15].

## Conclusions

In conclusion, we demonstrated that Ang II infusion is associated with kidney damage as well as increased SGLT2 cotransporter expression. The latter effect may represent an additional mechanism for Ang II to induce kidney damage. Therefore, blockade of the AT1 receptor prevents the cellular effects of Ang II including SGLT2-induced expression, preventing kidney damage. However, we also found that further inhibition of SGLT2, in the presence of an AT1 blockade, was associated with the additional prevention of kidney damage, probably by affecting tubuloglomerular feedback mechanisms or reactive oxygen species. Thus, the combination of AT1 receptor blockade with SGLT2 inhibition leads to a better prevention of Ang II-induced kidney damage compared with the effect of either AT1 receptor blockade or SGLT2 inhibition used as mono-therapy.

## Additional file

Additional file 1: **Figure S1.** Angiotensin II effect on Sodium-glucose cotransporter protein expression. LLC-PK_1_ cells (1 × 10^6^), were incubated during 24 h with increasing concentrations of Angiotensin II (10^-11^ to 10^-7^ M). Figures on left shows immunoblotting for control cells and cells incubated with Angiotensin II. Electrophoresis gels for immunoblot detection of SGLT2 and GAPDH were performed separately to avoid interference because their molecular weights are very close to each other. On right, figure shows blot analysis for each experimental condition. Each bar represents the mean ± SE of 5 different experiments, *p < 0.05 compared vs Control. (TIF 29990 kb)

## Data Availability

The datasets generated and analysed in the current study are available from the corresponding author on reasonable request.

## Abbreviations

Ang II or Ang: Angiotensin II
AT1: Angiotensin II receptor type 1
BBMV: Brush border membrane vesicles
BP: Blood pressure
DHE: Dihydroethidium;
Emp: Empagliflozin
GFR: Glomerular filtration rate
i.p: intraperitoneal
inulin-FITC: fluorescein isothiocyanate–inulin
Los: Losartan
PEG: Polyethylene glycol
ROS: Reactive oxygen species
SGLT2: Sodium-Glucose Cotransporter type 2
SOD: Superoxide dismutase
