# Supplementary figures and images for: Role of sodium/glucose cotransporter inhibition on a rat model of angiotensin II–dependent kidney damage
Source: BMC Nephrol. 2019 Aug 2;20:292. doi: 10.1186/s12882-019-1490-z (PMC6679465; doi:10.1186/s12882-019-1490-z)

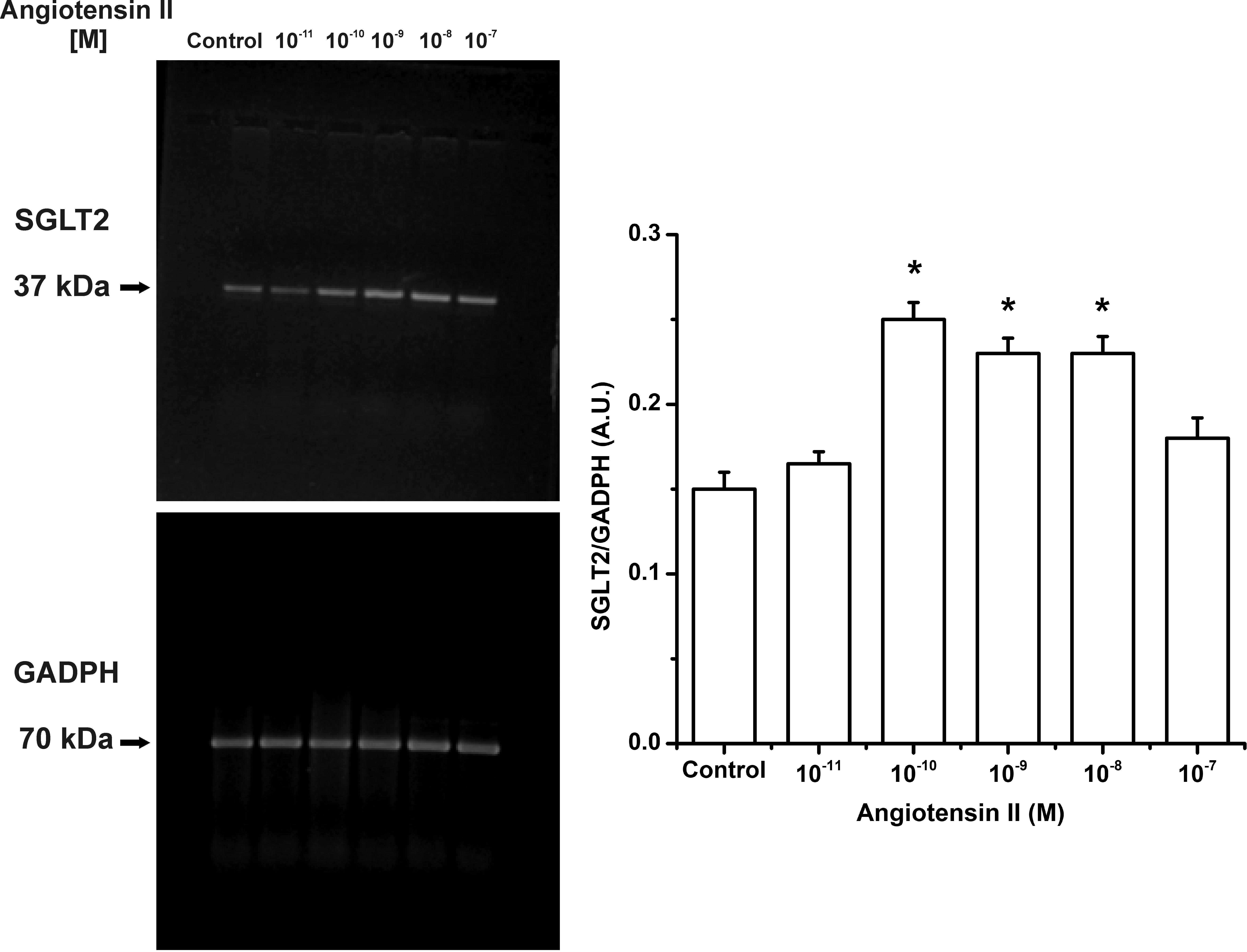

Supplement: Supplementary file 1 — Figure S1. Angiotensin II effect on Sodium-glucose cotransporter protein expression. LLC-PK1 cells (1 × 10^6^), were incubated during 24 h with increasing concentrations of Angiotensin II (10-11 to 10-7 M). Figures on left shows immunoblotting for control cells and cells incubated with Angiotensin II. Electrophoresis gels for immunoblot detection of SGLT2 and GAPDH were performed separately to avoid interference because their molecular weights are very close to each other. On right, figure shows blot analysis for each experimental condition. Each bar represents the mean ± SE of 5 different experiments, *p < 0.05 compared vs Control. (TIF 29990 kb) [file 12882_2019_1490_MOESM1_ESM.tif]
